# Determinants of sexual dysfunction among clinically diagnosed diabetic patients

**DOI:** 10.1186/1477-7827-9-70

**Published:** 2011-05-25

**Authors:** William KBA Owiredu, Nafiu Amidu, Huseini Alidu, Charity Sarpong, Christian K Gyasi-Sarpong

**Affiliations:** 1 Department of Molecular Medicine, School of Medical Sciences, College of Health Sciences, Kwame Nkrumah University of Science and Technology, Kumasi, Ghana; 2 Department of Medical Laboratory Technology, Faculty of Allied Health Sciences, College of Health Sciences, Kwame Nkrumah University of Science and Technology, Kumasi, Ghana; 3 Tema General Hospital, Tema, Greater Accra Region, Ghana; 4 Department of Surgery, (Urology Unit) Komfo Anokye Teaching Hospital/College of Health Sciences, Kwame Nkrumah University of Science and Technology, Kumasi, Ghana

## Abstract

**Background:**

Diabetes mellitus is a chronic disease that can result in various medical, psychological and sexual dysfunctions (SD) if not properly managed. SD in men is a common under-appreciated complication of diabetes. This study assessed the prevalence and determinants of SD among diabetic patients in Tema, Greater Accra Region of Ghana.

**Method:**

Sexual functioning was determined in 300 consecutive diabetic men (age range: 18-82 years) visiting the diabetic clinic of Tema General Hospital with the Golombok Rust Inventory of Sexual Satisfaction (GRISS) questionnaire, between November, 2010 and March, 2011. In addition to the socio-demographic characteristics of the participants, the level of glycosylated haemoglobin, fasting blood sugar (FBS) and serum testosterone were assessed. All the men had a steady heterosexual relationship for at least 2 years before enrolment in the study.

**Results:**

Out the 300 participants contacted, the response rate was 91.3% after 20 declined participation and 6 incomplete data were excluded All the respondents had at least basic education, 97.4% were married, 65.3% were known hypertensive, 3.3% smoked cigarettes, 27% took alcoholic beverages and 32.8% did some form of exercise. The 69.3% SD rate observed in this study appears to be related to infrequency (79.2%), non-sensuality (74.5%), dissatisfaction with sexual acts (71.9%), non-communication (70.8%) and impotence (67.9%). Other areas of sexual function, including premature ejaculation (56.6%) and avoidance (42.7%) were also substantially affected. However, severe SD was seen in only 4.7% of the studied population. The perceived "adequate", "desirable", "too short" and "too long intra-vaginal ejaculatory latency time (IELT) are 5-10, 5-10, 1-2 and 15-30 minutes respectively. Testosterone correlates negatively with glycated haemoglobin (HBA1c), FBS, perceived desirable, too short IELT, and weight as well as waist circumference.

**Conclusion:**

SD rate from this study is high but similar to that reported among self-reported diabetic patients in Kumasi, Ghana and vary according to the condition and age. The determinants of SD from this study are income level, exercise, obesity, higher perception of "desirable" and "too short" IELT.

## Background

Some of the consequences of diabetes include various medical [1], psychological [2], and sexual [3] dysfunctions. Among diabetic patients, hyperglycaemia can result in several complications ranging from short to long term effects. These complications could be avoided or deferred by effective control of the blood sugar level. Disorder of sexual function in men is a common under-appreciated complication of diabetes. SD among diabetic men may include disorders of libido, ejaculatory problems, and erectile dysfunction (ED). All three forms of SD can affect diabetic patients as well as their quality of life significantly. About 322 and 380 million people worldwide are projected to develop erectile dysfunction (ED) and diabetes respectively by the year 2025 with the largest projection increases in the developing countries [4,5].

The debate about the aetiology and risk factors for SD among diabetic patients is still on-going. Diabetic patients can develop both organogenic and psychogenic sexual dysfunction because they have a high likelihood of developing vascular and neurological complications as well as psychological problems [3]. As such, various efforts to elucidate the aetiology of SD among diabetic patients have suggested several factors (e.g. neurological, vascular, endocrine, and psychological) including the use of medication or a combined effect of some of these factors [6-8].

The prevalence of SD and diabetes varies widely probably because of the different definitions and the population studied, which in turn vary with respect to the number and selection of participants, cultural background, socioeconomic level, quality of psychosexual relationships and income. Few studies conducted among the Ghanaian community indicate 66% SD rate among the general male population [9], 59.8% among men with various medical conditions [10] and 59.2% among men in a marriage relationship [11] all domiciled in the Kumasi metropolis (Ashanti region). In the study among men with various medical conditions, those with self-reported diabetes had 70.0% SD rate [10]. However, it is not clear whether the prevalence of SD among the Ghanaian community would vary based on location and between clinically diagnosed and self-reported diabetic patients. Apart from these, the degree to which medical conditions and perceptual differences would affect SD is not known. Hence, this study assessed the prevalence as well as the determinants of SD among clinically diagnosed diabetic patients leaving in Tema, Greater Accra region of Ghana. The study also assessed what the participants considered to be normal and abnormal IELT. To our knowledge, this is the first study of SD conducted among this population in Ghana.

## Methods

### Participants

A cross-sectional study was conducted among 300 diabetic patients visiting Tema General Hospital in the Greater Accra region of Ghana. The Participants were recruited in a consecutive procedure from November 2010 to March 2011. Eligibility criteria for participants were as follows: sexually active, stable heterosexual relation for at least 2 years before enrollment in the study, aged 18 years or older and diabetic. A stable relationship was defined as one in which the man was engaged and maintains sexual relations, regardless of their marital status. The age range of the diabetic men involved in this study was between 18 and 82 years. Participation of the respondents was voluntary and informed consent was obtained from each participant. The study was approved by the Committee on Human Research, Publication and Ethics of the School of Medical Science and the Komfo Anokye Teaching Hospital, Kumasi.

### Procedure

All participants were evaluated by using a semi-structured questionnaire and the Golombok Rust Inventory of Sexual Satisfaction (GRISS).

### Socio-demographic and anthropometric data

A detailed self-designed semi-structured questionnaire was administered to each consented study participant for socio-demographic information including age, marital status, behavioural activities (smoking and alcohol consumption), educational background, occupation and income level. Body weight with study participants in light clothing was measured to the nearest 0.1 kg on a bathroom scale (Zhongshan Camry Electronics Co. Ltd. Guangdong, China) and height to the nearest 0.5 cm was measured with the study participants standing upright and barefooted, with the heels put together and the head in the horizontal plane against a wall-mounted ruler. Body mass index (BMI) was calculated by dividing weight (kg) by the height squared (m^2^). Waist circumference (to the nearest centimetre) was measured with a Gulick II spring-loaded measuring tape (Gay Mill, WI) midway between the inferior angle of the ribs and the suprailiac crest. Hip circumference was measured as the maximal circumference over the buttocks in centimeter and the waist to hip ratio (WHR) calculated by dividing the waist circumference (cm) by the hip circumference (cm).

### Measurements of perception of IELT

Questions regarding perception of normal and abnormal IELT were adapted and modified from a study among sex therapists conducted in the US and Canada [12]. The respondents were asked for background information (age, sex, occupation, educational level, marital status, etc.) and had questions about IELTs such as "too short," "adequate," "desirable," or "too long." The respondents were asked to give their opinion regarding, for example, "What is an *adequate *amount of time to elapse in sex from penile penetration of the vagina to ejaculation?" The question was asked in four different ways, with the italicized word changing from *adequate*, to *desirable*, to *too short*, to *too long*. This is an estimated time response, not a stop-watch-measured time response.

### The Golombok Rust Inventory of Sexual Satisfaction

Sexual response was measured by the Golombok Rust Inventory of Sexual Satisfaction (GRISS) questionnaire. The GRISS has 28 items on a single sheet and its use for assessing the existence and severity of sexual problems in heterosexual couples or individuals who have a current heterosexual relationship. All the 28 questions are answered on a five-point (Likert type) scale from "always", through "usually', "sometimes", and "hardly ever", to "never". It provides overall scores of the quality of sexual functioning within a relationship. In addition, subscale scores of impotence, premature ejaculation, infrequency, non-communication, dissatisfaction, non-sensuality and avoidance can be obtained and represented as a profile. Responses are summed up to give a total raw score (range 28-140). The total score and subscale scores are transformed using a standard nine point scale, with high scores indicating greater problems. Scores of five or more are considered to indicate SD. The GRISS was chosen because it is standardized, easy to administer and score, relatively unobtrusive and substantially inexpensive.

The GRISS can be used to assess improvement as a result of sexual or marital therapy and to compare the efficacy of different treatment methods. It can also be used to investigate the relationship between sexual dysfunction and extraneous variables. The subscales are particularly helpful in providing a profile for diagnosis of the pattern of sexual functioning within the couple, which can be of great benefit in designing a treatment program. The reliability of the overall scales has been found to be 0.94 for men and that of the subscales on average 0.74 (ranging between 0.61 and 0.83). Validity has been demonstrated under a variety of circumstances [13-15].

### Sample collection, preparation and analysis

Six milliliters (6 ml) of venous blood sample was collected from each participant in the morning between 07.00 to 09.00 GMT into Ethylene Diamine Tetraacetic Acid (EDTA) vacutainer^® ^tubes, Fluoride oxalate tube and evacuated gel tubes for serum preparation (Becton Dickinson, Rutherford, NJ). Samples in the EDTA tubes and fluoride oxalate tubes were used for HBA1 and fasting blood glucose measurement using BT 5000^® ^Random Access Chemistry Analyzer (Biotecnica, Italy) while samples in the evacuated gel tubes were centrifuged at 3000 *g *for 5 minutes and the serum aliquoted and stored in cryovials at a temperature of -80°C until time for testosterone assay using AxSYM automated analyzer (Abbott Diagnostics, USA). The AxSYM use Micro-particle Enzyme Immunoassay in the determination of Testosterone. The methods adopted by the automated instruments for the determination of biochemical parameters were according to the reagent manufacturers' instructions (JAS Diagnostics, Inc. Miami Florida, USA and Abbott Diagnostics, USA).

### Statistical analysis

The data were presented as mean ± SD or percentages. Logistic regression was used to assess the simultaneous influence of different variables in sexuality. In all statistical tests, a value of *p *< 0.05 was considered significant. The entry of the variables into the model was considered if p value is less than 0.05, and a stepwise procedure was applied. All analysis were performed using SigmaPlot for Windows, Version 11.0, (Systat Software, Inc. Germany) [16]

## Results

### Response rate, biochemical and socio-demographic characteristic

Out the 300 subjects interviewed, 20 refused to be part of this study leaving 280 respondents. Six (6) of the respondents had incomplete data leaving 274 evaluable data, giving a response rate of 91.3%. All the respondents had at least basic education with 39.1%, 15.7% and 10.6% having secondary, technical and tertiary education respectively. 97.4% of respondents were married, 65.3% were hypertensive, 3.3% smoked cigarettes, 27% took alcoholic beverages and 32.8% did some form of exercise.

The mean age, weight, BMI and income level of the study population was 59.9 ± 11.3 years, 76.0 ± 14.3 kg, 26.8 ± 9.8 kg m^-2 ^and Ghc 212.9 ± 200.6 respectively from the socio-demographic characteristic in Table 1. When the study population was stratified based on SD, those with SD were significantly older (p < 0.0001), heavier (p = 0.0078 for weight and p = 0.0462 for BMI) and had higher income level (p = 0.0033) as compared to those without SD (Table 1).

**Table 1:** General characteristic of the study population stratified by sexual dysfunction

Variables	Total	NSD	SD	P value
***Socio-demographic data***				
Age (yrs)	59.9 ± 11.3	57.2 ± 11.6	66.1 ± 7.5	< 0.0001
Weight (kg)	76.0 ± 14.3	72.6 ± 10.3	77.6 ± 15.5	0.0078
Height (m)	1.7 ± 8.2	1.7 ± 7.1	1.7 ± 8.6	0.1840
BMI (kg m^-2^)	26.8 ± 9.8	25.5 ± 3.0	26.6 ± 4.5	0.0462
Income level (Ghc)	212.9 ± 200.6	159.7 ± 81.5	236.6 ± 231.4	0.0033
***Perceived intra-vaginal ejaculatory latency time***				
Adequate (min.)	8.2 ± 4.7	7.4 ± 2.5	8.8 ± 5.3	0.0275
Desirable (min.)	8.5 ± 4.9	7.7 ± 2.6	9.1 ± 5.6	0.0259
Too short (min.)	1.6 ± 1.4	1.3 ± 0.7	1.7 ± 1.5	0.0101
Too long (min.)	24.2 ± 10.9	25.5 ± 8.1	23.7 ± 11.9	0.2046
***Biochemical data***				
FBS (mmol L^-1^)	9.4 ± 4.0	9.3 ± 3.4	9.4 ± 4.2	0.8317
Testosterone (ng mL^-1^)	6.3 ± 2.5	6.7 ± 2.8	6.0 ± 2.1	0.0250
HBA1c (%)	8.6 ± 1.9	8.7 ± 2.1	8.6 ± 1.8	0.7354
***Sexual dysfunction subscales***				
Impotence	5.2 ± 2.0	3.4 ± 1.6	6.2 ± 1.4	< 0.0001
Premature ejaculation	4.9 ± 1.8	3.4 ± 0.9	5.6 ± 1.7	< 0.0001
Non-sensuality	5.1 ± 1.9	3.3 ± 1.7	5.8 ± 1.3	< 0.0001
Avoidance	4.9 ± 1.8	4.9 ± 2.4	4.9 ± 1.5	0.9507
Dissatisfaction	5.0 ± 1.8	3.5 ± 1.9	5.7 ± 1.4	< 0.0001
Non-communication	5.0 ± 1.9	3.3 ± 1.6	5.7 ± 1.5	< 0.0001
Infrequency	5.2 ± 1.8	4.9 ± 1.8	5.3 ± 1.8	0.0874

***Data are presented as mean ± Std. Dev. Participant without sexual dysfunction (NSD) were compared with those with sexual dysfunction (SD) using unpaired t-test***.

The mean testosterone level was significantly lower (p = 0.0250) when those with SD (6.0 ± 2.1 ng mL^-1^) was compared to those without SD (6.7 ± 2.8 ng mL^-1^). However, the mean stanine scores as derived from the various SD subscales were significantly higher among those with SD as compared to those without SD as shown in Table 1.

### Sexual function-GRISS

**Figure 1:**
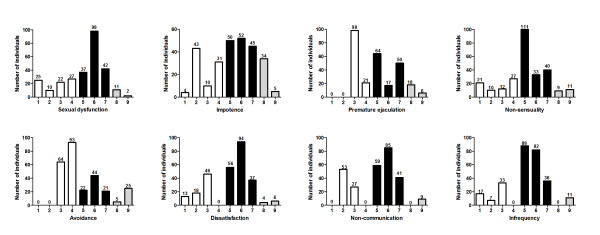
**Scores of sexual dysfunction in 292 studied population according to GRISS questionnaire**. Graph shows the distribution of scores (from 1 to 9 on the x- axis) for each GRISS subscale, with the number of patients (y-axis) above each score. Normal scores are 1 to 4 (clear columns), abnormal scores are 5 to 9 (shaded columns) and severe abnormal scores are 8 and 9 (grey columns).

The sexual function scores of the participants for each GRISS subscale are shown in Figure 1. All the respondents had one or more subscale scores reflecting sexual problems (score of 5 or above). The prevalence of SD among the respondents in this study is 69.3% (i.e. 190 out of 274). The most prevalent areas of difficulty were infrequency (217 of 274, 79.2%), non-sensuality (204 out of 274, 74.5%), dissatisfaction with sexual acts (197 of 274, 71.9%), non-communication (194 of 274, 70.8%), impotence (186 out of 274, 67.9%), premature ejaculation (155 out of 274, 56.6%) and avoidance (117 out of 274, 42.7%).

However, severe SD was seen in 4.7% of the studied population (i.e. 13 out 274). Also the most prevalent areas of severe difficulty were impotence with (39 of 274, 14.2%), avoidance (30 out of 274, 10.9%), premature ejaculation (24 out of 274, 8.8%), non-sensuality (20 out of 274, 7.3%), infrequency (11 out of 274, 4.0%), dissatisfaction (10 out of 274, 3.6%) and non-communication (9 out of 274, 3.3%) (Figure 1).

### Risk factors

The effect of different socio-demographic variables on the SD risk is recorded in Table 2. Higher income level (OR = 2.1; 95% CI = 1.0-4.3; p = 0.042 for Ghc 111-400 and OR = 14.3; 95% CI = 1.7-119.7; p = 0.014 for Ghc > 400), exercise (OR = 2.2; 95% CI = 1.3-3.7; p = 0.004) and obesity (OR = 11.9; 95% CI = 2.7-52.8; p = 0.001) were the variables that significantly increased the risk of SD from the univariate analysis. Also, in Table 3, those who perceived desirable IELT higher than 13 min. were 10 times more likely to have SD as compared to those who perceived 7 to 13 min. as being "desirable" IELT (OR = 10.1; 95% CI = 1.3-77.9; p = 0.026) and those who perceived IELT greater than 2 min. as being too short are also at about 13 times more likely to develop SD as compared to those who perceived 1 to 2 min. as being too short (OR = 13.3; 95% CI = 1.8-99.9; p = 0.012) (Table 3).

**Table 2:** Rate of sexual dysfunction according to socio-demographic risk factors

Variables	n/N*	Rate of SD (%)	OR(95% CI)	P value	**aOR(95% CI**)	P value
***Marital status***						
Married	185/267	69.3	0.9(0.2-4.7)	0.904	1.7(0.2-15.3)	0.628
Single	5/7	71.4				
***Educational level***						
Basic	68/95	71.6				
Secondary	71/107	66.4	0.78(0.43-1.4)	0.424	0.5(0.3-1.1)	0.087
Technical	27/43	62.8	0.67(0.31-1.45)	0.303	0.6(0.3-1.5)	0.283
Tertiary	24/29	82.8	1.91(0.7-5.5)	0.234	1.5(0.5-5.1)	0.464
***Income level***						
No income	4/13	30.8	2.1(0.5-7.7)	0.309	2.9(0.7-12.5)	0.152
< 111	19/36	52.8				
111-400	146/208	70.2	2.1(1.0-4.3)	0.042	2.1(1.0-4.7)	0.048
>400	16/17	94.1	14.3(1.7-119.7)	0.014	21.7(2.4-197.7)	0.006
***Smoking***						
Yes	7/9	77.8	1.6(0.3-7.7)	0.580	1.4(0.2-9.0)	0.707
No	183/265	69.1				
***Alcohol***						
Yes	48/74	64.9	0.8(0.4-1.3)	0.329	0.9(0.5-1.7)	0.774
No	142/200	71.0				
***Exercise***						
No	54/93	58.1				
Yes	136/181	75.1	2.2(1.3-3.7)	0.004	2.0(1.1-3.6)	0.023
***Body weight***						
Underweight	12/15	80.0	2.3(0.6-8.9)	0.220	2.3(0.6-9.4)	0.249
Normal	50/79	63.3				
Overweight	87/137	63.5	1.0(0.6-1.8)	0.975	0.7(0.4-1.3)	0.240
Obese	41/43	95.3	11.9(2.7-52.8)	0.001	10.4(2.3-47.6)	0.003

*Number of subjects with SD/number of subjects in each category

**Table 3:** Rate of sexual dysfunction according to perceived intra-vaginal ejaculatory latency time, testosterone and glycated haemoglobin

Variables	n/N*	Rate of SD (%)	**OR(95% CI**)	P value	**aOR(95% CI**)	P value
***Adequate***						
Low	1/1	100	NA		NA	
Normal	100/144	69.4				
High	89/129	69.0	1.0(0.6-1.6)	1.000	0.4(0.2-1.2)	0.121
***Desirable***						
Low	85/125	68.0	1.1(0.6-1.8)	0.787	1.4(0.8-2.5)	0.296
Normal	85/128	66.4				
High	20/21	95.2	10.1(1.3-77.9)	0.026	4.1(1.1-41.3)	0.038
***Too short***						
Low	25/37	67.6	1.1(0.5-2.2)	0.870	1.1(0.4-2.5)	0.893
Normal	139/210	66.2				
High	26/27	96.3	13.3(1.8-99.9)	0.012	8.2(1.0-74.1)	0.040
***Too long***						
Low	3/3	100.0	NA		NA	
Normal	174/257	67.7				
High	13/14	92.9	6.2(0.8-48.2)	0.081	2.3(0.2-26.7)	0.500
***Testosterone***						
Low	6/12	50.0	0.4(0.1-1.3)	0.122	0.3(0.1-1.2)	0.077
Normal	161/225	71.6				
High	23/37	62.2	0.7(0.3-1.3)	0.249	0.9(0.4-2.0)	0.775
***HBA1c***						
Normal	17/27	63.0				
High	173/247	70.0	1.4(0.6-3.1)	0.450	1.0(0.4-2.6)	0.947

*Number of subjects with SD/number of subjects in each category

After adjusting for confounding factors which includes age, the risk factors for SD are higher income level, exercise, obesity, perception of desirable IELT greater than 13 min and perception of too short IELT greater than 2 min. (Table 2 and 3).

### Perception of IELT

The questions of primary interest in the measurement of perceived IELT involved respondent's definitions of "adequate" and "desirable" IELTs. The mean ± SD for these variables were, respectively, 8.2 ± 4.7 and 8.5 ± 4.9 minutes, with interquartile ranges (IQRs), respectively, of 5.0 to 10.0 (median = 7.0) and 5.0 to 10.0 (median = 8.0) minutes. (The IQR represents the responses of the middle 50% of respondents, the range from the 25^th ^percentile to the 75^th ^percentile of responses). The respondents were also asked the definitions for IELTs that were "too short" or "too long". The mean ± SD for these were, respectively, 1.6 ± 1.4 and 24.2 ± 10.9 minutes; IQRs for these variables were, respectively, 1.0 to 2.0 (median = 1.0) and 15.0 to 30.0 (median = 30.0) minutes (Table 1). However, when the perceived IELT were classified based on SD, those with SD significantly perceived higher time as being "adequate" (8.8 ± 5.3 min.), "desirable" (9.1 ± 5.6 min.) and "too short" (1.7 ± 1.5 min.) as compared to those without SD (7.4 ± 2.5, 7.7 ± 2.6 and 1.3 ± 0.7 for "adequate", "desirable" and "too short" respectively) (Table 1).

Overall, about half of the studied population perceived "adequate" and "desirable" IELT to last for 3-7 and 7-13 minutes respectively, while about 80% and 90% perceived "too short" and "too long" IELT to last 1-2 and 10-30 minutes respectively. About 47%, 8%, 10% and 5% perceived "adequate", "desirable", "too short" and "too long" IELT to last more than 7, 13, 2 and 30 minutes respectively (Table 4). When the perception was stratified based on sexual function, higher proportion of those with SD think that more than 13 minutes, 2 minutes and more than 30 minutes as being desirable (10.5%), too short (13.7%) and too long (6.8%) IELT respectively compared to 1.2% each for desirable, too short and too long among those without SD. Conversely, significantly lower proportion of those with SD perceived too short and too long IELT to last 1-2 minutes and 10-30 minutes respectively (Table 4).

**Table 4:** Prevalence of abnormal perception of intra-vaginal ejaculatory latency, testosterone and glycated haemoglobin stratified by sexual dysfunction

Variables	Total (n = 274)	NSD (n = 84)	SD (n = 190)	P value
***Adequate (3-7)***				
Low	1(0.4%)	0(0.0%)	1(0.5%)	0.5053
Normal	144(52.6%)	44(52.4%)	100(52.6%)	0.9694
High	129(47.1%)	40(47.6%)	89(46.8%)	0.9054
***Desirable (7-13)***				
Low	125(45.6%)	40(47.6%)	85(44.7%)	0.6587
Normal	128(46.7%)	43(47.1%)	85(44.7%)	0.3235
High	21(7.7%)	1(1.2%)	20(10.5%)	0.0074
***Too short (1-2)***				
Low	37(13.5%)	12(14.3%)	25(13.2%)	0.8012
Normal	210(76.6%)	71(84.5%)	139(73.2%)	0.0403
High	27(9.9%)	1(1.2%)	26(13.7%)	0.0014
***Too long (10-30)***				
Low	3(1.1%)	0(0.0%)	3(1.6%)	0.2469
Normal	257(93.8%)	83(98.8%)	174(91.6%)	0.0222
High	14(5.1%)	1(1.2%)	13(6.8%)	0.0501
***Testosterone (2.25-9.72)***				
Low	12(4.4%)	6(7.1%)	6(5.1%)	0.1372
Normal	230(83%)	66(78.6)	164(86.3)	0.1074
High	32(11.7%)	12(14.3%)	20(10.5%)	0.3717
***HBA1c (3-6)***				
Low	0(0.0%)	0(0.0%)	0(0.0%)	NA
Normal	27(9.9%)	10(11.9%)	17(8.9%)	0.4489
High	247(90.1%)	74(88.1%)	173(91.1%)	0.4489

### Relationships between variables

Age generally associate positively with SD as well as its subscales. For the purpose of interpretation, Cohen [17] considered 0.10 <*r *< 0.30 as small, 0.30 <*r *< 0.50 as medium and r *>*0.50 as large. SD increase with increase income level, greater perception of desirable and too short IELT. The degree of impotency also increase with increase income level, increase exercise level, increased perception of adequate, desirable, too short IELT and decreased testosterone level. Premature ejaculation is directly linked with increased exercise and higher perception of too short IELT in this study (Table 5). Non-sensuality correlate positively with income level, desirable and too short IELT whilst avoidance is positively associated with smoking and FBS but negatively with higher perception of "adequate", "desirable" and "too long" IELT. The lower the levels of sexual satisfaction from this study the higher the perception of "adequate", "desirable" and "too short" IELT. Non-communication is positively linked with income levels and higher perceptions of what was "adequate" IELT (Table 5).

**Table 5:** Partial correlation between sexual dysfunction parameters and socio-demographic data, perceived intra-vaginal ejaculation latency time, as well as biochemical data

Variables	SD	IMP	PE	NS	AV	DIS	NC	INF
Age	0.39***	0.31***	0.33***	0.39***	0.15**	0.24***	0.21***	0.06
Education	-0.04	0.10	-0.07	-0.09	-0.07	0.05	0.05	0.00
Income level	0.20**	0.21***	0.10	0.15*	-0.02	0.05	0.13*	0.03
Smoking	0.04	-0.09	0.04	0.00	0.15*	-0.01	-0.02	0.10
Alcohol	-0.03	-0.03	-0.09	0.00	0.09	0.02	-0.05	0.03
Exercise	0.08	0.12**	0.13*	0.06	-0.08	0.02	-0.03	-0.06
Adequate	0.10	0.15**	0.03	0.07	-0.13*	0.16**	0.15*	-0.07
Desirable	0.13*	0.19**	0.08	0.13*	-0.15*	0.15**	0.03	-0.10
Too short	0.16**	0.17**	0.13*	0.16**	-0.11	0.20**	0.02	-0.07
Too long	-0.09	0.00	-0.08	0.00	-0.19**	-0.06	0.02	-0.09
BMI	0.01	0.07	-0.04	-0.07	0.02	-0.09	-0.01	0.02
FBS	0.06	0.05	0.04	0.04	0.16**	-0.07	0.00	0.09
Testosterone	0.05	-0.14*	0.01	0.09	-0.03	0.06	0.09	0.00
HBA1c	0.00	0.06	-0.04	-0.06	0.07	0.06	0.04	0.05

*Correlation is significant at the 0.05 level (2-tailed), **Correlation is significant at the 0.01 level (2-tailed), ***Correlation is significant at the 0.001 level (2-tailed). Boldface r = **Pearson product moment correlation coefficient with a medium size (0.30 ≤ *r≥ = *0.50) effect**.

Generally, SD is linked positively with all the subscales. The subscales are also related positively with each other except for a negative association between premature ejaculation and avoidance as well as between avoidance and non-communication (Table 6).

**Table 6:** Pearson Product Moment Correlation Coefficient between sexual dysfunction including the 7 subscales of the GRISS

Variables	IMP	PE	NS	AV	DIS	NC	INF
Sexual dysfunction	0.76***	0.69***	0.74***	0.14*	0.63***	0.62***	0.16**
Impotence (IMP)		0.46***	0.54***	-0.04	0.49***	0.39***	-0.01
Premature ejaculation (PE)			0.54***	-0.18**	0.38***	0.37***	-0.06
Non-sensuality (NS)				-0.09	0.44***	0.47***	-0.10
Avoidance (AV)					-0.02	-0.13*	0.25***
Dissatisfaction (DIS)						0.39***	0.02
Non-communication (NC)							0.01

*Correlation is significant at the 0.05 level (2-tailed), **Correlation is significant at the 0.01 level (2-tailed), ***Correlation is significant at the 0.001 level (2-tailed). Boldface r = **Pearson product moment correlation coefficient with a medium size (0.30 ≤ = *r *≥*= *0.50) effect: boldface and underline *r ***= **Pearson product moment correlation coefficient **with **a large size *(r ***> 0**.50) effect, INF = Infrequency**.

As shown in Table 7, testosterone correlates negatively with HBA1c, FBS, perceived desirable, too short IELT, and weight as well as waist circumference. Glycated haemoglobin correlates positively with perceived adequate, desirable, too long IELT and FBS. The older the study participant, the lower the income level, exercise level, perceived adequate, desirable IELT and FBS but the higher the WC. Those with higher educational level had higher income level, smoked less cigarette and perceived less time as being too short IELT. Cigarette also correlates positively with alcohol consumption. The perception of IELT as well as markers of obesity correlates positively with each other with a small to a large size effect (Table 7).

**Table 7:** Pearson Product Moment Correlation Coefficient between biochemical, socio-demographic and perceived IELT variables

Variables	HBA1c	Age	Edu	Income	Smk	Alc	Exr	**Adeq**.	**Des**.	TS	TL	WT	BMI	WC	WHR	FBS
Testosterone	-0.12*	-0.07	-0.08	-0.02	-0.09	-0.07	-0.09	-0.11	-0.12*	-0.16*	-0.06	-0.23***	0.02	-0.14*	-0.05	-0.12*
HBA1c		-0.07	-0.02	-0.07	-0.04	-0.06	-0.10	0.18**	0.13*	-0.01	0.17**	-0.03	0.03	-0.04	0.02	0.31***
Age			0.02	-0.16**	0.06	-0.06	-0.14*	-0.15*	-0.21***	-0.11	-0.09	-0.10	0.06	0.14*	0.02	-0.21***
Education (Edu)				0.24***	-0.16**	-0.03	0.07	-0.05	-0.09	-0.17**	-0.04	0.07	0.03	0.08	-0.02	0.02
Income					0.05	-0.07	0.02	-0.02	-0.01	0.00	-0.07	0.04	0.00	-0.01	-0.03	-0.02
Smoking (Smk)						0.14*	-0.04	-0.05	-0.05	-0.04	-0.08	-0.04	-0.01	-0.04	-0.01	0.02
Alcohol (Alc)							0.00	0.09	0.04	0.00	0.03	-0.03	0.03	-0.09	-0.05	0.03
Exercise (Exr)								-0.10	-0.03	-0.02	-0.10	0.18**	-0.01	0.02	0.00	-0.20***
Adequate (Adeq.)									0.83***	0.63***	0.60***	0.15*	0.02	0.06	-0.05	0.03
Desirable (Des.)										0.73***	0.66***	0.21***	0.04	0.07	-0.04	-0.02
Too short (TS)											0.34***	0.16*	0.05	0.09	-0.05	-0.09
Too long (TL)												0.12	0.06	0.03	0.00	-0.05
Weight (WT)													0.15*	0.42***	-0.01	-0.17**
Body mass index (BMI)														0.43***	-0.01	-0.08
Waist circumference (WC)															0.01	-0.09
Waist to Hip Ratio (WHR)																0.02

*Correlation is significant at the 0.05 level (2-tailed), **Correlation is significant at the 0.01 level (2-tailed), ***Correlation is significant at the 0.001 level (2-tailed). Boldface r = **Pearson product moment correlation coefficient with a medium size (0.30 ≤ = *r *≥*= *0.50) effect: boldface and underline *r ***= **Pearson product moment correlation coefficient **with **a large size *(r ***> 0**.50) effect, FBS = Fasting Blood Sugar**

## Discussion

According to the World Health Organization, SD is defined as "the various ways in which an individual is unable to participate in a sexual relationship as he or she would wish". Diabetes mellitus could lead to multiple medical [1], psychological [2], and sexual [3] dysfunctions. Reduced sexual function is a well-documented complication of diabetes. Previous reports have shown that diabetic men are at increased risk for SD at an earlier age [7,8,18,19], with an incidence ranging from 20% to 85% [18,20,21]. Most of the risk factors for SD (such as vascular disease, hypertension, peripheral neuropathy and obesity) overlap with many of the comorbidities linked with diabetes with prevalence and severity being more common in people with diabetes than in the general population [22].

The 69.3% rate of SD observed among this cohort of diabetic men was higher than the 66% reported among the general Ghanaian male population [9], 59.8% reported among Ghanaian men with various medical conditions [10] and the 59.2% reported among men in a marriage relationship [11]. However, this figure (69.3%) is in agreement with the 70.0% reported among self-reported diabetic subjects [10]. The agreement between the SD rate among self-reported and the clinically diagnosed diabetics in this study is reasonable since it can be assumed that subjects who reported that they were 'diabetic' did so on the basis of medical diagnosis. In all these studies, SD rate increased with age. High rate of SD among diabetic subjects could be due to the fact that, as part of the complications associated with diabetes, there is damage to small arteries and arterioles which could impair endothelium-dependent relaxation of penile smooth muscle thus preventing optimal blood flow to and from the penis, and maintenance of an erection [22,23]. Further research will however be required in Ghana to determine the prevalence rate of SD among type 1 and 2 diabetics and whether there are any differences in their association with SD.

In contrast, the 69.3% is higher than the 37% reported among Hong Kong diabetic men [19] and the 63.6% reported among Chinese diabetic men [24] but agrees with the 20% to 85% incidence rate for diabetic subjects [18,20,21] reported in other studies. This wide variation in the incidence of SD could be due in part to the definition used for SD, the period of data retrieval, the population surveyed, the setting in which the patients were studied, the manner in which the participants were questioned, the number and selection of participants, cultural background, socioeconomic level, quality of psychosexual relationships and income. Apart from these, the degree to which a medical condition and perceptual differences would affect SD is not known.

The observed higher perception of what is "adequate", "desirable" and "too short" IELT among those with SD from this study coupled with the positive association of perceived IELT with SD, impotence, premature ejaculation and dissatisfaction means that these groups of diabetic men are unable to satisfy their sexual needs probably due to their perception of IELT. The innate standards as well as belief of an individual as modified by the type of formal and informal education received from the society, including pornographic movies could affect the quality of life and leads to distress and displeasure. Since stereotype and not reality is the main determinant of expectation [25], dissatisfaction due to wrong perception may ultimately lead to the purchase of performance enhancing medication even when such an individual does not actually need it, as observed currently among Ghanaian men (Amidu, personal observation). Care should be taken not to diagnose these groups of men as having PE. Recently, men who are not satisfied with their IELT while having a normal or even long IELT duration, have been classified as Premature-like Ejaculatory Dysfunction [26]. According to Waldinger *et al.*, this PE subtype has a clearly different aetiology and pathogenesis than lifelong PE or acquired PE [27]. These Ghanaian cohorts of men are most likely the group of men who have Premature-like Ejaculatory Dysfunction.

The intersection between perceived "adequate" (5.0 to 10.0 minutes) and "desirable" (5.0 to 10.0 minutes) IELT means that an IELT of 5 to 10 minutes is perceived by respondents as normal. From this study, there is positive association between the perceived "too short" IELT and PE. For those who are dissatisfied with their ejaculation time, it may be erroneously classified as premature ejaculation, when their actual ejaculation time is less than their desirable IELT or the IELT that is generally considered as adequate in this population. The significant direct effect of perceived IELT with the level of dissatisfaction with sexual intercourse is in agreement in part with the finding of Patrick *et al.*, [28] who reported that, IELT has a significant direct effect on perceived control over ejaculation, but not a significant direct effect on ejaculation-related personal distress or satisfaction with sexual intercourse [28]. Self-estimated IELT is normally adequate for assessing PE in everyday clinical practice despite the fact that self-estimated and stopwatch-measured IELT are interchangeable and correctly assigned PE status with 80% sensitivity and 80% specificity [29].

The higher perception of "desirable" and "too short" IELT being a predictor of SD could account for the high rate of SD amongst subjects with higher expectations, thus further education and sensitization is needed to educate people on what adequate and too short IELT entails. This will go a long way to ease expectations, restore confidence and eventually eliminate inadequacy and gradually restore sexual function to normalcy. The boom of advertisement of sex enhancing drugs in the media does not help the situation and people are made to feel that sexual longevity is necessary to satisfy their partners and thus establishing a vicious cycle of inadequacy, lower perceptions of performance and eventually SD.

Several studies have demonstrated SD in diabetic populations, but the nature of the sexual complaints among this group is limited mainly to erectile dysfunction. As indicated by the GRISS, it appears that SD in this study is mainly related to infrequency (79.2%), non-sensuality (74.5%), dissatisfaction with sexual acts (71.9%), non-communication (70.8%) and impotence (67.9%). Other areas of sexual function, including premature ejaculation (56.6%) and avoidance (42.7%) were also substantially affected. However, severe SD was seen in 4.7% of the studied population. Also the most prevalent areas of severe difficulty were impotence with (14.2%), avoidance (10.9%), premature ejaculation (8.8%), non-sensuality (7.3%), infrequency (4.0%), dissatisfaction (3.6%) and non-communication (3.3%).

The reduction in testosterone level among the participants with SD is in agreement with previous reports [30-33]. Testosterone could enhance copulation via increases in dopamine release in the medial preoptic area, perhaps through up-regulation of NO synthesis [34,35]. Androgens have long been implicated in the regulation of sexual behaviour in the human male [36]. Higher testosterone levels could shorten the latency of erection activated by the introduction to sexual material [37], and testosterone substitution in hypogonadal males rejuvenates sexual interest, decreases latency, and increases frequency and enormity of nocturnal penile tumescence (NPT) [38]. Available data also support the negative association of testosterone with markers of glycaemic control and obesity as shown by this study [39-43]. Increase in visceral, central or abdominal adiposity as measured by WC and possibly weight can lead to endocrinologic imbalances. These have been shown to relate positively with insulin, glucose levels and negatively with testosterone levels [39]. This study is also in agreement with the assertion that WC should be the preferred anthropometric variables in predicting endogenous testosterone level [39-43]. The mechanism could be due in part to increase in serum leptin level production [44] and/or excess cortisol secretion [45] which mimic LH/hCG-stimulated androgen suppressing androgenic hormone formation.

From this study, it seems that higher level of education offer the participants' better job and income level. Participants with SD had higher income levels and were heavier as compared to those without sexual dysfunction. High income and obesity were found to be risk factors for SD from this study. It is not very surprising to find this phenomenon in Africa (at least in Ghana) because the higher class of African societies with higher income levels are known to be the major consumers of junk food, alcohol and in developing stress free lifestyles which are basically sedentary, whilst the poor and low income earners struggle to feed well and are exposed to strenuous activity. Even though obesity was not a significant risk factor for SD in our previous report among the general male populace as well as those with various medical conditions [9,10], it could mean that the impact of obesity on the sexual function of the diabetic patient is different from that observed among non-diabetic subjects. Obesity is associated with a state of chronic oxidative stress and inflammation [46] leading to the impairment of endothelial function resulting in SD and laying the ground work for atherosclerosis [47]. Since atherosclerosis of the arteries supplying genital tissues greatly affects sexual function, it seems rational to assume that conditions predisposing to atherosclerosis (diabetes, obesity) might impair sexual function.

Reported literature indicates a decreased relative risk of developing SD with increased physical activity [48,49]. Whether this reduced risk applies to diabetic men is not known. Exercise from this study was a significant risk factor even after adjustment for age, income levels and obesity. This finding is contrary to our previous report among the general male population [9] and among men with various medical conditions [10] where exercise was not a significant risk factor for SD. Reasons for this disparity is not readily known from this study, however, in a follow-up study by Derby *et al.*, [50], overweight men at baseline were found to be at an increased risk of developing SD regardless of whether they lost weight. Even though exercise is a key aspect of a healthy lifestyle, strenuous physical exercise results in increased oxygen consumption, increased metabolism and increased production of reactive oxygen species which would ultimately lead to oxidative stress [51]. Diabetes has also been thought to be mediated by oxidative stress as the underlying mechanism. Thus as diabetics exercise they confound their oxidative stress levels and this will eventually worsen their health and cause sexual dysfunction. There is therefore the need for further study to define the level of exercise needed by diabetic patients for effective glycaemic control and also to prevent oxidative stress induced SD.

## Conclusion

The prevalence of SD (69.3%) among these diabetic patients is high but similar to that reported among self-reported diabetic patients (70.0%) in Kumasi, Ghana and correlates positively with age, income level and perceived desirable and too short IELT. The determinants of SD from this study are income level, exercise, obesity, higher perception of "desirable" and "too short" IELT. The perceived "adequate", "desirable", "too short" and "too long IELT are 5-10, 5-10, 1-2 and 15-30 minutes respectively. This could impact significantly on the individual's self-esteem and quality of life thereby causing emotional distress leading to relationship problems.

## Competing interests

The authors declare that they have no competing interests.

## Authors' contributions

NA and WKBAO developed the concept and designed the study. NA, WKBAO, HA, CS and CKG-S administered the questionnaire, assay for FBS, HBA1c and testosterone, analysed and interpreted the data. NA, HA, CS and CKG-S drafted the manuscript. NA, WKBAO, HA, CS and CKG-S revised the manuscript for intellectual content. All authors read and approved the final manuscript.
